# Porous Single‐Crystal Nitrides for Enhanced Pseudocapacitance and Stability in Energy Storage Applications

**DOI:** 10.1002/advs.202410429

**Published:** 2024-11-08

**Authors:** Xiangqi Gao, Guoliang Ma, Cong Luo, Shaobo Xi, Lingting Ye, Kui Xie

**Affiliations:** ^1^ Key Laboratory of Design & Assembly of Functional Nanostructures Fujian Institute of Research on the Structure of Matter Chinese Academy of Sciences Fuzhou Fujian 350002 China; ^2^ Fujian College University of Chinese Academy of Sciences Fuzhou Fujian 350002 China; ^3^ University of Chinese Academy of Science Beijing 100049 China; ^4^ School of Mechanical Engineering Shanghai Jiao Tong University 800 Dongchuan Road Shanghai 200240 China

**Keywords:** Nb_4_N_5_, porous single crystal, pseudocapacitance, Ta_3_N_5_

## Abstract

Supercapacitors have emerged as a prominent area of research in energy storage technology, primarily because of their high power density and notable stability compared to batteries. However, their practical implementation is hindered by their low energy densities and insufficient long‐term stability. In this study, bulk porous Nb_4_N_5_ and Ta_3_N_5_ single crystals with excellent pseudocapacitance and electrical conductivity are successfully prepared by solid‐phase transformation method. These monolithic porous single crystals (PSC) exhibit a long‐range ordered crystalline architecture and substantial specific surface area, which facilitate rapid charge transport and ion diffusion within the electrolyte‐permeated crystal lattice. Notably, the areal capacitance of the porous Nb_4_N_5_ single crystals is 12.9 F cm^−2^ at a current density of 6 mA cm^−2^ and 35.08 F cm^−2^ at a scan rate of 1 mV s^−1^. Furthermore, the energy density reached 1.79 mWh cm^−2^ at a power density of 20 mW cm^−2^, demonstrating their high energy storage capability. Moreover, these porous Nb_4_N_5_ single crystals exhibited robust capacitance retention and exceptional cycling stability, making them promising candidates for use as electrodes in energy storage applications. These results underscore the significant potential of porous metal nitride single crystals in advancing the field of capacitive energy storage.

## Graphical abstract

This study develops centimetre‐scale porous nitride single crystals, integrating single‐crystal stability with high surface area. Their well‐ordered lattice structure and interconnected porosity significantly enhance the capacitance and durability of supercapacitor electrodes. The findings underscore the potential of transition metal nitrides exhibiting high pseudocapacitance and long‐term stability, thereby advancing the development of efficient and robust pseudocapacitance electrodes.

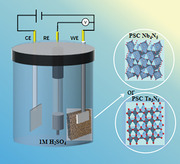

## 1 Introduction

Rapid technological developments have made innovations in energy storage technology crucial for the advancement of electric vehicles, wearable devices, smart grids, and other fields. The development of energy storage devices is fundamental to ensuring the stable utilisation of renewable energy.^[^ ^1^, ^2^, ^3^ ^]^ Pseudocapacitance has attracted considerable interest owing to its high power density, high energy density, rapid charging and discharging capabilities, and other advantages. However, the energy densities of pseudocapacitors are significantly lower than those of batteries.^[^ ^4^, ^5^, ^6^, ^7^, ^8^, ^9^ ^]^ The main challenges associated with these materials are their low energy density, which results from their low capacitance, and poor stability caused by the irreversibility of the redox process during charge‐discharge cycling.^[^ ^10^, ^11^, ^12^, ^13^ ^]^ Capacitor energy density is affected by a variety of factors, including electrode material, electrolyte, capacitor structure, and other factors such as temperature and manufacturing process.^[^ ^14^ ^]^ Oxide electrodes,^[^ ^15^, ^16^ ^]^ such as ruthenium dioxide and manganese dioxide, are the most widely studied pseudocapacitive electrodes and exhibit good capacitance properties. Ruthenium dioxide electrodes offer good electrical conductivity and high capacitance; however, their high cost limits their application. Manganese dioxide electrodes suffer from low electrical conductivity and poor chemical stability in electrolytes, which significantly affects the enhancement of their capacitance and stability during energy storage. Cobalt oxide electrodes are another type of material with high pseudocapacitance, demonstrating good corrosion resistance and stability; however, their capacitance is insufficient for practical applications.^[^ ^17^ ^]^

Transition‐metal nitrides with high capacitance have been extensively studied in recent years.^[^ ^16^, ^18^, ^19^ ^]^ Their high conductivity and good pseudocapacitance properties make them promising materials for energy storage. Conventional nitride electrodes are typically fabricated using nanomaterials, such as nanoparticles,^[^ ^20^ ^]^ nanowires,^[^ ^21^, ^22^ ^]^ and nanofilms,^[^ ^23^ ^]^ to enhance their specific areal capacitance. However, the low stability of nanomaterials and the low conductivity caused by contact resistance in nanostructured electrodes reduce their capacitance and stability. Therefore, the growth of bulk metal nitride materials with high specific areal capacitance is a promising strategy for enhancing capacitance. PSC nitrides have emerged in recent years and have demonstrated great potential in the field of supercapacitors.^[^ ^24^, ^25^ ^]^ Their porous structure increases the specific surface area of the electrode material, thereby providing more active sites for charge storage and transport. Furthermore, the effective shortening of ion and electron transport paths enhances the charge and discharge rates of the electrode. In addition, PSC exhibit high conductivity, good mechanical strength, and corrosion resistance owing to their unique crystal structures and excellent chemical stabilities, making them ideal electrode materials.

In this study, we prepared centimetre‐scale porous nitride single crystals that integrate the high stability of single‐crystal materials with the highly exposed active sites of porous materials. We grew bulk PSC Nb_4_N_5_ and PSC Ta_3_N_5_ from NaNbO_3_ and KTaO_3_ single crystals, demonstrating that their highly exposed, long‐range ordered active sites can enhance pseudocapacitance and cycling stability.^[^ ^24^ ^]^ The uniformly ordered lattice structure and disordered interconnected pores of these materials can significantly enhance the capacitance and stability of supercapacitor electrodes.^[^ ^25^ ^]^ Owing to the unsaturated coordination of Nb‐N and Ta─N on the exposed surface of the porous nitride, the active sites promote reversible redox reactions during chemisorption and desorption processes.^[^ ^26^ ^]^ The porous structure, which includes diffusion channels for ions in the electrolyte and a skeleton for electron transformation, has the potential to significantly enhance capacitance properties.^[^ ^27^ ^]^ In addition, Nb_4_N_5_ and Ta_3_N_5_ exhibit good chemical stability in acid electrolytes. The uniform single‐crystal structure also reduces electron scattering, resulting in good electronic conduction properties.^[^ ^28^ ^]^ Therefore, PSC nitrides exhibit excellent stabilities because of their single‐crystal properties.

## 2 Physicochemical and Structural Characteristics

**Figure 1:**
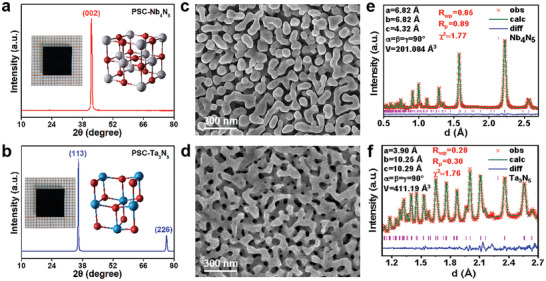
a,c,e) XRD patterns, SEM images and NPD Rietveld refinement patterns of PSC Nb_4_N_5_ monoliths; b,d,f)XRD patterns, SEM images and NPD Rietveld refinement patterns of PSC Ta_3_N_5_ monoliths.

In this study, we employed a lattice reconstruction technique to grow PSC Nb_4_N_5_ by nitriding NaNbO_3_ single crystals under an ammonia gas flow rate of 100–350 mL min^−1^ at 780–950 °C and a pressure of 50–760 Torr. Figure S1 (Supporting Information) shows a scanning electron microscope (SEM) image of the nitriding process of the NaNbO_3_ single crystal into PSC Nb_4_N_5_. The growth interface between NaNbO_3_ and Nb_4_N_5_ is clearly visible, indicating solid‐state growth of the single crystal from the surface to the interior. Figure S2 (Supporting Information) shows the X‐ray diffraction (XRD) patterns and crystal structure maps, demonstrating the transition from the NaNbO_3_ single crystal to PSC Nb_4_N_5_ and revealing the underlying growth mechanism. The results suggest that as Na and O atoms evaporate, N diffuses into the lattice and replaces O atoms, leading to a localised contraction of the lattice that promotes the recombination growth of PSC Nb_4_N_5_. **Figure** **1**a shows the XRD pattern of PSC Nb_4_N_5_ with (002) facet surfaces, demonstrating its single‐crystal characteristics. A photograph of a 1 cm PSC Nb_4_N_5_ with a thickness of 1 mm is also shown. Figure 1c illustrates the microstructure of the PSC Nb_4_N_5_ oriented toward the (002) facet, revealing earthworm‐like connecting pores that confirm its porous structure. We performed Rietveld refinement on the neutron powder diffraction (NPD) spectra of Nb_4_N_5_ and Ta_3_N_5_, as shown in Figure 1e,f, respectively. The R_wp_, R_P_, and χ^2^ values of the PSC Nb_4_N_5_ sample are 0.85%, 0.89%, and 1.77, respectively, which align well with the experimental data. These experimental and computational results indicate that the sample possessed a tetragonal structure with a space group of *I4/m*. The calculated cell parameters for the sample are a = b = 6.82 Å and c = 4.32 Å. These smaller cell parameters can be attributed to the higher content of nitrogen vacancies in the samples prepared under vacuum, which resulted in a larger ionic radius of the Nb ions.

Similarly, PSC Ta_3_N_5_ was prepared using a lattice reconstruction strategy at temperatures of 800–1000 °C, under a pressure of 50–760 Torr, with an NH_3_ flow rate of 200–400 mL min^−1^. The (111)‐oriented KTaO_3_ single crystal was transformed into (113)‐oriented PSC Ta_3_N_5_, as confirmed by the XRD patterns shown in Figure S3a,b (Supporting Information). The growth mechanism is shown in Figure S3c,d (Supporting Information). Figure 1b shows the XRD spectra of PSC Ta_3_N_5_ with the (113) facet, demonstrating its single‐crystal properties, along with a photograph of a PSC Ta_3_N_5_ measuring 1 cm in size and 1 mm in thickness. Figure 1d shows the SEM morphology of PSC Ta_3_N_5_ with the orientation of (113) plane. As shown in Figure 1f, the R_wp_, R_P_, and χ^2^ values of the PSC Ta_3_N_5_ sample are 0.28%, 0.30%, and 1.76, respectively, and the experimental and computational results indicate that the sample has an orthorhombic structure with a space group of *Cmcm*. The cell parameters of the sample were calculated to be a = 3.90 Å, b = 10.25 Å and c = 10.29 Å. The EDS results in Figure S4 (Supporting Information) show that the molar ratios of the metal atoms to nitrogen for Nb_4_N_5_ and Ta_3_N_5_ were 4:5 and 3:5, respectively. Figure S5 (Supporting Information) presents the Raman spectra of the NaNbO_3_ and KTaO_3_ parent crystals, along with PSC Nb_4_N_5_ and PSC Ta_3_N_5_ obtained through nitriding.

The porosities of PSC Nb_4_N_5_ and PSC Ta_3_N_5_ were 43% and 53%, respectively. As shown in Figure S6 (Supporting Information), the specific surface areas of PSC Nb_4_N_5_ and PSC Ta_3_N_5_ were 5.86 and 5.24 m^2^ g^−1^, with pore sizes of 40.5 and 43.62 nm, respectively. The porous structure increased the specific surface area of the electrode material, thereby providing more active sites for charge storage and transport. Furthermore, it effectively shortened the ion and electron transport paths, thereby improving the charge/discharge rates of the electrodes. Both PSC Nb_4_N_5_ and PSC Ta_3_N_5_ were prepared using a lattice reconstruction method, suggesting that this approach may be effective for synthesizing other porous nitrides.

**Figure 2:**
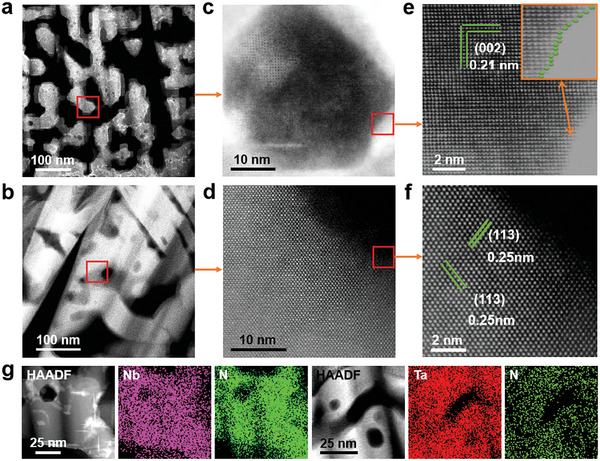
STEM of PSC Nb_4_N_5_ and PSC Ta_3_N_5_; a,b) Monocrystalline skeleton; c–f) STEM images and HRTEM images of twisted surfaces and lattice structures; g) Mapping of two PSC materials.

The spherical aberration corrected transmission electron micrograph (STEM) images of PSC Nb_4_N_5_ and PSC Ta_3_N_5_ are shown in **Figures** **2** and S7 (Supporting Information). Figure S7a,b (Supporting Information) illustrate the microscopic morphology of PSC Nb_4_N_5_ and PSC Ta_3_N_5_ after slicing the surface layer at random locations using a focused ion beam. The internal pores of both PSC types exhibit worm‐like configurations and are distributed relatively uniformly. Figure 2a,b illustrates magnified views of the local skeleton pore map at a scale of 100 nm. Despite the greater pore distribution, the single‐crystal skeleton remained stable. Figure 2c,d show random regions where the high‐resolution transmission electron microscopy (HRTEM) images reveal the distribution of atomic arrangements. Figure 2e shows a crystal spacing d of 0.21 nm, which corresponds to the PSC Nb_4_N_5_ (002) facet. Figure 2f shows crystal spacing d of 0.25 nm, which correspond to the (113) facet of PSC Ta_3_N_5_.

Single‐crystal diffraction scanning was conducted using the selected area electron diffraction mode of STEM to determine the crystal structure and assess single‐crystallinity in specific regions. Figure S7e,f (Supporting Information) shows regularly aligned diffraction spots and diffracted crystal planes that satisfy vector superposition, indicating that PSC Nb_4_N_5_ and PSC Ta_3_N_5_ exhibit single‐crystal regularity in their alignment. Combined with the presence of disordered pores in the material, this suggests that PSC Nb_4_N_5_ and PSC Ta_3_N_5_ are novel single‐crystal‐like materials with microscopically localized ordering. The distorted surface of the hole is shown in the box in Figure 2c. Figure 2d provides a high‐resolution local zoom revealing that, despite slight lattice distortions around the hole, the lattice orientation remained coherent. The distance between neighbouring Nb atoms was ≈0.297 nm, resulting in the formation of an Nb‐Nb metallic bond and demonstrating the typical metallic state and electronic conduction behaviour. Given that the porous structures introduce discontinuities within the crystal structure, an unsaturated Nb‐N coordination structure should be present within the pores. Figure S8 (Supporting Information) shows the electronic states of the fine structure of the twisted surface, revealing a deficiency of electrons on the distorted surface and a clear charge transfer from the Nb/Ta atoms to the lower N atoms. This transfer resulted from the coordination of Nb/Ta atoms with lower N atoms, thereby forming an active structure on the continuously distorted surface. This active structure enhanced the interaction between the adsorbed substances and the well‐defined surface structure. The elemental mapping results in Figure 2g show that Nb/Ta and N were uniformly distributed in PSC Nb_4_N_5_ and PSC Ta_3_N_5_.

**Figure 3:**
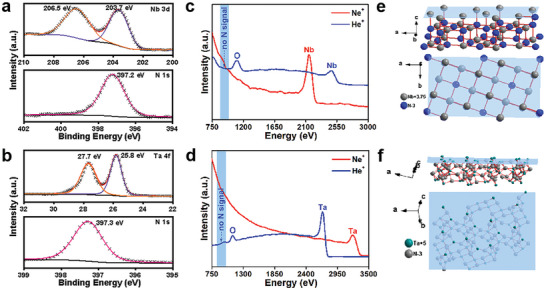
a,b) XPS of PSC Nb_4_N_5_ and PSC Ta_3_N_5_, respectively; c,d) the HS‐LEISS of the outermost surface layer of the two PSC; e,f) are the orientation diagrams of the two crystals.

The X‐ray photoelectron spectroscopy (XPS) results in **Figure** **3**a,b shows the interaction between Nb/Ta and N, with Nb‐N and Ta─N bonds acting as intermediates between ionic and covalent bonds.^[^ ^29^ ^]^ The presence of mixed‐valence cations (Nb^3+^ and Nb^5+^) not only generates Faraday pseudocapacitance but also leads to good metal‐like conductivity. Figure S9 (Supporting Information) shows the bandgaps and densities of states of PSC Nb_4_N_5_ and PSC Ta_3_N_5_. As shown in Figure S9a (Supporting Information), PSC Nb_4_N_5_ exhibits negligible bandgap and conductive characteristics with metallic properties. As shown in Figure S9b (Supporting Information), PSC Ta_3_N_5_ has a bandgap of 1.24 eV, indicating that it is a narrow‐band‐gap semiconductor. The conduction band of PSC Nb_4_N_5_ is primarily composed of the 4d orbitals of Nb atoms, whereas the conduction band of PSC Ta_3_N_5_ predominantly comprises the 5d orbitals of Ta atoms, which are the primary source of its electrical conductivity. In both materials, the valence band is predominantly formed by the 2s and 2p orbitals of the N atoms. As shown in Figure 3c,d, the high sensitivity low energy ion scattering spectroscopy (HS‐LEISS) results confirm that the atomic termination surfaces are Nb atoms. Ne ion scattering of the outermost atoms of PSC Nb_4_N_5_ identified Nb, while He ion impacts revealed the presence of oxygen due to the oxidisability of nitrides; however, no N signals were detected at the spectral positions. Similarly, PSC Ta_3_N_5_ shows Ta as the termination layer, confirming that PSC Ta_3_N_5_ is also highly unsaturated with nitrogen coordination; therefore, unsaturated metal‐nitrogen coordination likely exists between the outermost metal Nb/Ta atoms and their attached nitrogen atoms.

Figure 3e,f shows simulations of the crystal surface. The metal atoms are distributed on the outermost surface of PSC Nb_4_N_5_, while the N atoms are located in the spacers of the Nb atoms.^[^ ^30^ ^]^ Because the N atoms were not present on the surface of the single crystal, the unsaturated coordination of the Nb‐N bonds at the surface was enhanced. This unsaturated nitrogen coordination is further confirmed by the electron paramagnetic resonance spectra of PSC Nb_4_N_5_ and PSC Ta_3_N_5_, as shown in Figure S10 (Supporting Information). The g coefficient, which was ≈2, can be attributed to the unpaired electrons in the Nb‐N/Ta─N bonds on the unsaturated coordination surface of the PSC.^[^ ^31^, ^32^, ^33^, ^34^ ^]^ As shown in Figure S11 (Supporting Information), the differential charge of the Nb‐N coordination structure reveals electron‐deficient active centres and significant charge transfer from the Nb atoms on the surface. This transfer of electrons from the metal to the nitrogen atoms results from the differing electronegativities of the metal‐nitrogen bonds.

## 3 Electrochemical Evaluation

**Figure 4:**
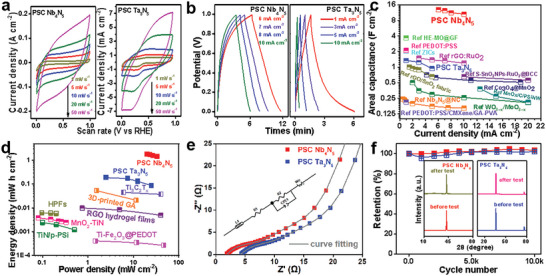
Electrochemical performance of PSC Nb_4_N_5_ and PSC Ta_3_N_5_ in 1 M H_2_SO_4_ electrolyte. a) the CV curves; b) the GCD curves; c) Areal capacitance; d) Ragone plots; e) Nyquist plots; f) CV cycling diagrams.

**Figure** **4**a shows the cyclic voltammetry (CV) curves of the PSC Nb_4_N_5_ and PSC Ta_3_N_5_ electrodes in a 1 m H_2_SO_4_ solution at various scan rates. The nearly rectangular CV curves indicate that the two electrode materials exhibit nearly ideal capacitance characteristics, reflecting their excellent charge propagation abilities. The high electrical conductivity and porous microstructure of the electrodes facilitated the diffusion of the substance during charging and discharging, resulting in high current densities and a broad voltage window in this CV profile. The areal capacitance of PSC Nb_4_N_5_ and PSC Ta_3_N_5_ reached 35.08 F cm^−2^ (108.2 F g^−1^) and 1.79 F cm^−2^ (5.18 F g^−1^), respectively, at a scan rate of 1 mV s^−1^. The areal capacitance of PSC Nb_4_N_5_ was significantly higher than that of conventional pseudocapacitive materials. Figure 4b shows the galvanostatic charge‐discharge (GCD) curves of the two electrode materials in a 1 m H_2_SO_4_ solution at different currents. The potential window for both charging and discharging was 1 V. The triangular GCD curves show that both electrode materials exhibited good resistivity and reversibility during the energy storage process. The areal capacitances of both electrode materials at different currents, as shown in Figure 4c were compared with those of materials reported in other studies.^[^ ^5^, ^35^, ^36^, ^37^, ^38^, ^39^, ^40^, ^41^, ^42^, ^43^, ^44^, ^45^, ^46^ ^]^ The areal capacitance, which was calculated based on the GCD curves, reached 12.9 F cm^−2^ (39.8 F g^−1^) for PSC Nb_4_N_5_ at a current of 6 mA cm^−2^, surpassing those of the other materials. In contrast, at a current of 1 mA cm^−2^, the areal capacitance of PSC Ta_3_N_5_ was 1.35 F cm^−2^ (3.91 F g^−1^). The main reason for this lower capacitance value is the low conductivity of PSC Ta_3_N_5_, which results in a slower charge‐transfer rate and greater energy loss during the charge‐discharge process. Figure 4d shows the Ragone plots for PSC Nb_4_N_5_ and PSC Ta_3_N_5_ and compares their energy and power densities with those reported in the literature. PSC Nb_4_N_5_ achieved an energy density of 1.79 mWh cm ^−2^ at 6 mA cm^−2^ and maintained an energy density of 1.47 mWh cm^−2^ at 10 mA cm^−2^. PSC Ta_3_N_5_ achieved an energy density of 0.19 mWh cm^−2^ at a current density of 1 mA cm^−2^ and maintained 0.09 mWh cm^−2^ at 10 mA cm^−2^. These results further demonstrate that both electrode material devices exhibited excellent multiplier performance. Moreover, these energy density values are significantly higher than those of recently reported all‐solid‐state asymmetric supercapacitor devices.^[^ ^47^, ^48^, ^49^, ^50^, ^51^, ^52^, ^53^, ^54^ ^]^

Unsaturated coordination creates active surfaces (primarily dominated by Nb‐N_1/5_, Nb‐N_2/5_ and Nb‐N_3/5_), which can reversibly adsorb and desorb H^+^, resulting in pseudocapacitance.^[^ ^1^ ^]^ To elucidate the mechanisms of chemisorption on twisted surfaces, we simulated hydrogen adsorption on a coordination‐unsaturated Nb/Ta─N structure within the inner surface of the pores, as shown in Figure S12 (Supporting Information). The Nb‐N_1/5_, Nb‐N_2/5_ and Nb‐N_3/5_ sites on the twisted surface of PSC Nb_4_N_5_ exhibited the average adsorption energy of ≈−0.85 eV for H atoms. The average adsorption energy of captured H atoms on the twisted surface of PSC Ta_3_N_5_ was ≈−1.15 eV. Figure S13 (Supporting Information) shows that the adsorption energies of H atoms at Nb‐N_1/5_, Nb‐N_2/5_ and Nb‐N_3/5_ range from ≈−0.6 to −1.3 eV, indicating a reasonable energy barrier for reversible chemisorption. To explore the energy storage mechanism of the PSC electrode, we used in situ Raman spectroscopy to monitor its structural changes during charging, as shown in Figure S14 (Supporting Information), where an enhancement of the intensity of the Raman characteristic peaks is seen by increasing the voltage due to the obvious coupling between the vibrational modes and electronic excitation. The desorption of hydrogen atoms after the loss of electrons leads to a change in the intramolecular resonance frequency, which in turn affects the position of the peaks in the Raman spectrum. The breaking of hydrogen or chemical bonds leads to a weakening of the vibrational frequency of the molecule, which in turn leads to a red shift in the Raman spectrum. The charge storage mechanism of PSC relies on the storage of charge through rapid surface redox reactions. The pseudocapacitive behaviour depends on the trapping of H atoms within the coordination‐unsaturated structure of the distorted surface in the pores, as along with the redox reactions of electrons within the crystal structure. During charging, H^+^ is extracted from PSC Nb_4_N_5_, whereas during discharge, H^+^ is captured in the coordination‐unsaturated Nb‐N structure. These charging and discharging processes are related to the redox reactions of H^+^ and the gain and loss of electrons. PSC Ta_3_N_5_ operates via the same mechanism. In Figure S15 (Supporting Information), we analyze the correlation between current density and scan rate with fitted b‐values of 0.83 and 0.75, respectively, which suggests the predominance of surface‐controlled capacitance processes and that the capacitance properties originate from chemisorption on the electrode surface.^[^ ^37^, ^55^ ^]^

Figure 4e shows the electrochemical impedance spectra recorded over the frequency range of 0.1–100 kHz. The bulk solution resistance (*Rs*) of PSC Nb_4_N_5_ was measured to be as low as 1.85 Ω, indicating high electrical conductivity. The absence of impedance arcs in the high‐frequency region suggests that the rapid diffusion of species within the porous structure of PSC Nb_4_N_5_ can be attributed to its high specific surface area, intrinsic electrical conductivity, and electrochemical activity. In addition, the homogeneity of the single‐crystal‐like structure reflects the ordering of the active sites, which enhances both pseudocapacitance and long‐term stability. The *Rs* of PSC Ta_3_N_5_ reached up to 4.3 Ω, which is significantly higher than that of PSC Nb_4_N_5_. In conductive PSC Nb_4_N_5_, rapid charge transfer and significantly reduced energy loss resulted in high capacitance during energy storage. Figure 4f shows the cycling performance stability of PSC Nb_4_N_5_ and PSC Ta_3_N_5_. CV was conducted at a sweep rate of 100 mV s^−1^. Both materials exhibited no significant degradation in performance after 10000 cycles during the charging and discharging processes, indicating that PSC Nb_4_N_5_ and PSC Ta_3_N_5_ have high stability during energy storage. As shown in the inset, XRD analysis of the materials before and after testing revealed no notable shift in the positions of the peaks. This suggests that the test process did not alter the structural properties of the materials, thereby validating their structural stability. SEM after the reaction, as shown in Figure S16 (Supporting Information), showed no significant change in porosity or morphology, again reflecting the stability of the material structure and its resistance to fatigue during cycling.

## 4 Conclusion

In conclusion, we prepared PSC Nb_4_N_5_ and PSC Ta_3_N_5_ using a lattice reconstruction transformation strategy. Both materials exhibited with high specific surface areas and good resistivities, making them ideal electrodes for pseudocapacitors. PSC Nb_4_N_5_ features unsaturated metal‐nitrogen coordination and a uniform single‐crystal structure with ordered active sites, which promote rapid and reversible redox reactions, thereby enhancing pseudo‐capacitance. Its capacitance reached 12.9 F cm^−2^ in a 1 m H_2_SO_4_ solution, with no degradation observed after 10000 cycles. The PSC Ta_3_N_5_ electrode exhibited a capacitance of 1.35 F cm^−2^ and excellent stability owing to its porous and single‐crystal structure. Considering their high activity, conductivity and stability transition‐metal nitrides are promising materials for pseudocapacitor energy storage.

## Conflict of Interest

The authors declare no conflict of interest.

## Supporting information



Supporting Information

## Data Availability

The data that support the findings of this study are available from the corresponding author upon reasonable request.
