# Supplementary material for: Porous Single‐Crystal Nitrides for Enhanced Pseudocapacitance and Stability in Energy Storage Applications
Source: Adv Sci (Weinh). 2024 Nov 8;12(1):2410429. doi: 10.1002/advs.202410429 (PMC11714193; doi:10.1002/advs.202410429)
Supplement: Supplementary file 1 — Supporting Information [file ADVS-12-2410429-s001.docx]

**Porous Single-Crystal Nitrides for Enhanced Pseudocapacitance and Stability in Energy Storage Applications**

Xiangqi Gao^1,2,3^, Guoliang Ma^1,2^, Cong Luo^1,2^, Shaobo Xi^1,2^, Lingting Ye^1,2,^*, Kui Xie^1,2,3,4,^*

^1^ Key Laboratory of Design & Assembly of Functional Nanostructures, Fujian Institute of Research on the Structure of Matter, Chinese Academy of Sciences, Fuzhou, Fujian, 350002, China

^2^ Fujian College, University of Chinese Academy of Sciences, Fuzhou, Fujian, 350002, China

^3^ University of Chinese Academy of Science, Beijing, 100049, China

^4^ School of Mechanical Engineering, Shanghai Jiao Tong University, 800 Dongchuan Road, Shanghai, 200240, China

E-mail: [ltye@fjirsm.ac.cn](mailto:ltye@fjirsm.ac.cn); [xiekui@sjtu.edu.cn](mailto:xiekui@sjtu.edu.cn)

**Experimental Procedure:**

1. **Crystal growth.**

The grown NaNbO_3_ single crystal by the molten salt method is polished and cut into a 10 mm $\times$10 mm$\times$1 mm substrate, and then processed in aluminum oxide tube under 50-760 Torr pressure in ammonia atmosphere at 780-950 °C by chemical vapor deposition system to grow PSC Nb_4_N_5_.

The grown KTaO_3_ single crystal is polished and cut into a 10 mm $\times$10 mm$\times$1 mm substrate, and then processed in aluminum oxide tube under 50-760 Torr pressure in ammonia atmosphere at 800-1000 °C by chemical vapor deposition system to grow PSC Ta_3_N_5_.

**2. Characterization method.**

We use XRD (Mniflex 600, Panalytical) to characterize the phase of the generated material to confirm whether it is a single crystal phase. The flat samples are placed on the FE-SEM (SU-8010) sample platform to characterize the micro channels, and the EDS and Mapping are used to characterize the element analysis and distribution. TEM samples were prepared using focused ion beam (FIB) nano-tomography (Helios 650, Zeiss Auriga) and the lattice structure was observed using Cs-TEM (FEI Titan3 G2 60-300).The chemical states of elements are determined by XPS (ESCALAB 250Xi). Raman spectra were obtained by Labram HR evolution Raman spectroscopy (Horiba Jobin Yvon). The neutron scattering patterns are collected at the Dongguan neutron spallation source multiphysics (MPI) spectrometer. High sensitivity low energy ion scattering spectroscopy (HS-LEISS, Qtac100, ION-TOF) was used to examine the atomic termination layers of the samples. Electron paramagnetic resonance (EPR) spectra were acquired using a spectrometer (Bruker Biospin GMBH E500 10/12) to identify nitrogen vacancies.

**3. Experimental test.**

All electrochemical experimental tests such as CV, GCD and long-term cycle were conducted using the electrochemical workstation (IM6 Zahner, Germany). The electrochemical impedance spectroscopy (EIS) was recorded in the frequency range of 0.1-100 kHz. The area of PSC Nb_4_N_5_ and PSC Ta_3_N_5_ used for the test is 0.15 cm^2^, 0.2 cm^2^ respectively, and the thickness is 1mm.

4. Theoretical calculations

Density Functional Theory (DFT) calculations using the Vienna Ab Initio Simulation Package (VASP).^[1]^ The generalized gradient approach (GGA) is used to simulate exchange-correlation interactions, including the Perdew-Burke-Ernzerhof (PBE) function,^[2]^ and the projection-enhanced wave (PAW) method is applied to show the interactions between the core and valence electrons. The energy and residual force of Nb_4_N_5_ converged to 10^-5^ eV and 0.02 eV A ^-1^ during the electronic and geometric optimization. The optimized lattice parameters of the Nb_4_N_5_ crystal are a = 6.936 Å, b = 6.936 Å, and c = 4.4327 Å, and the k-point lattice is 5 × 5 × 8. The plane-wave cutoff energy is 500 eV. The p (2×2) superstructure of Nb_4_N_5_ (001), (100), and (010) surfaces is used to simulate the periodic plate model. The 20 Å vacuum region is used in these models to avoid the interaction between the plate and its periodic image. A 3 × 3 × 1 K-point grid is used in the model of Brillouin region sampling. The energy and residual force of Ta_3_N_5_ converged to 10^-4^ eV and 0.02 eV A ^-1^ during electronic and geometric optimization. The lattice parameters of the optimized Ta_3_N_5_ crystal are a = 3.909 Å, b = 10.301 Å, c = 10.301 Å, and the K- point is 10 × 3 × 3. The plane wave cutoff energy is 500 eV. The adsorption energy is defined as : E_ads_ = (E_Ad-surface_ − E_surface_ − nE_Ad_)/n, where E_Ad-surface_ is the total energy of the adsorbate interacting with the surface, and E_surface_ is the energy of clean crystal surface; E_Ad_ is the energy of free adsorbate in the gas phase; n is the number of adsorbates.


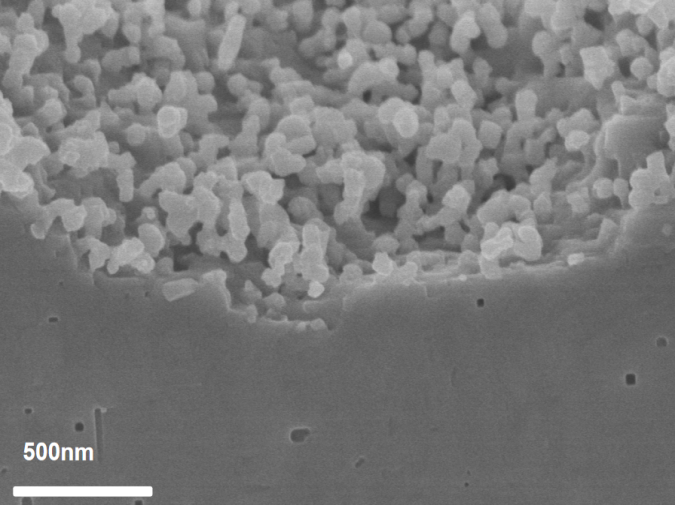


**Figure S1.** SEM image of NaNbO_3_ reaction for 15 h.


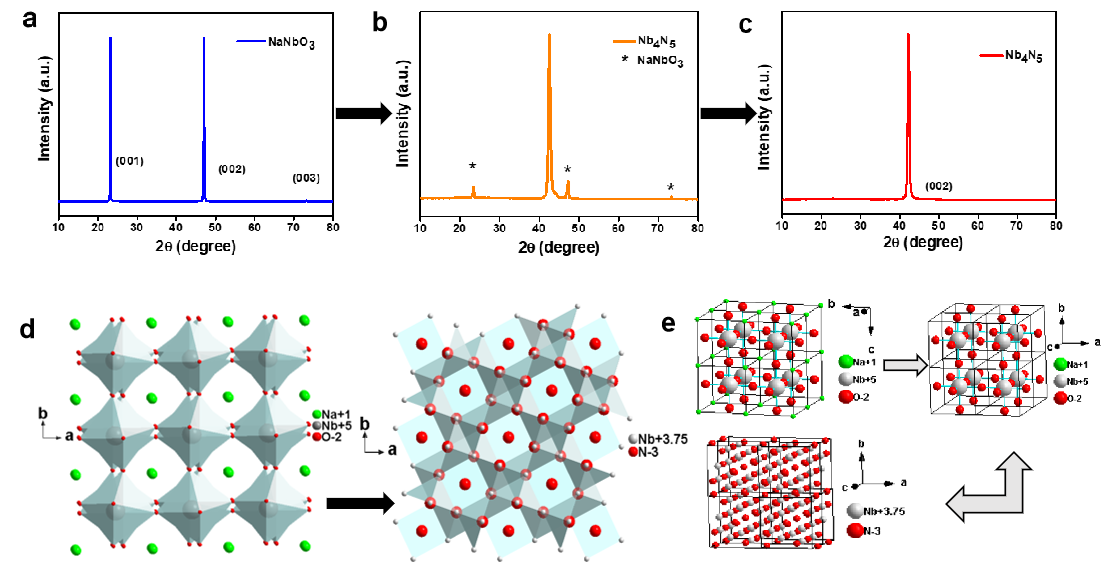


**Figure S2.** XRD and the formation process of PSC Nb_4_N_5_. (a) (001) NaNbO_3_ facet, (b) intermediate state of NaNbO_3_ and Nb_4_N_5_, (c) (001) facet of Nb_4_N_5_. (d) The supercell polyhedral model growth process of the porous single crystal from the (001) facet of NaNbO_3_ to the (002) facet of Nb_4_N_5,_ and (e) the corresponding ball-and-stick model diagram of this process.


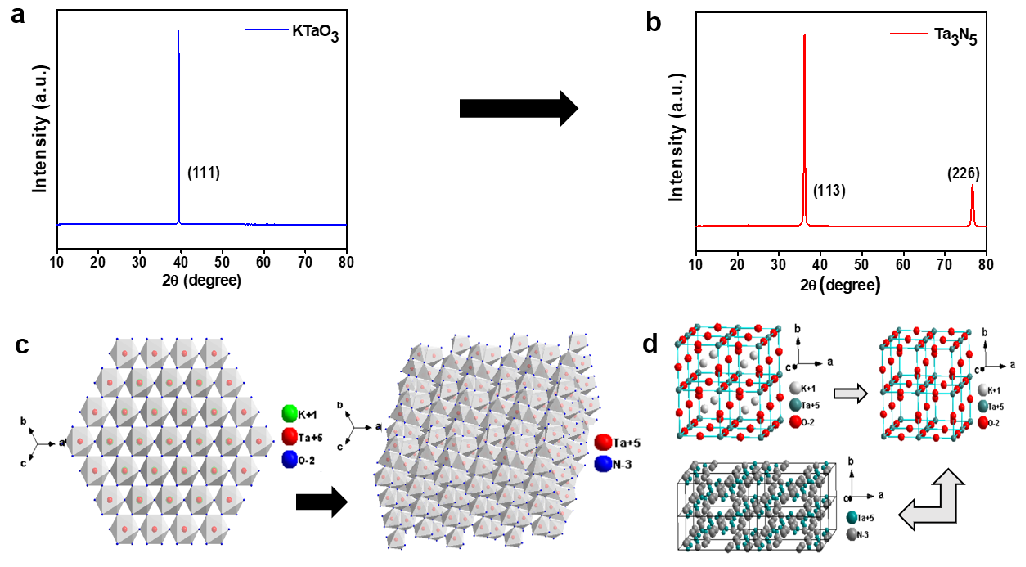


**Figure S3.** XRD and the formation process of PSC Ta_3_N_5_. (a) (111) plane of KTaO_3_ and (b) (113) plane of Ta_3_N_5_. (c) The growth process of the supercell polyhedral model of the porous single crystal from the (111) facet of KTaO_3_ to the (113) facet of Ta_3_N_5_, and (d) the ball-and-stick model of this process.

**
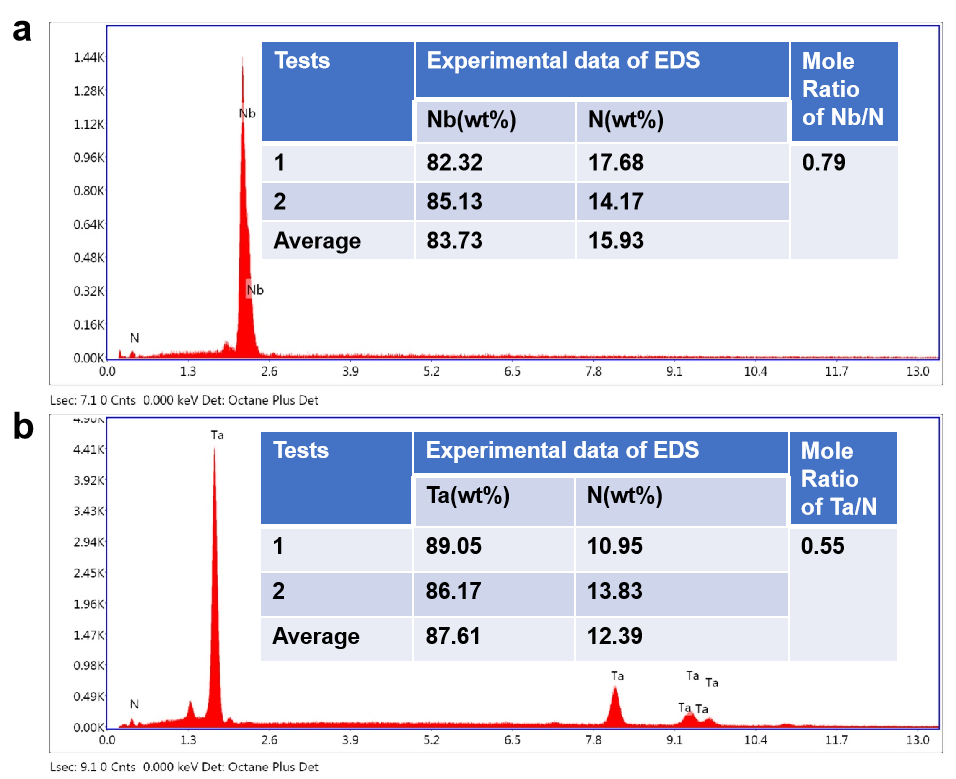
**

**Figure S4.** EDS of PSC Nb_4_N_5_ and PSC Ta_3_N_5_. (a, b) EDS of the (002) face of Nb_4_N_5_ and the (113) face of Ta_3_N_5_.


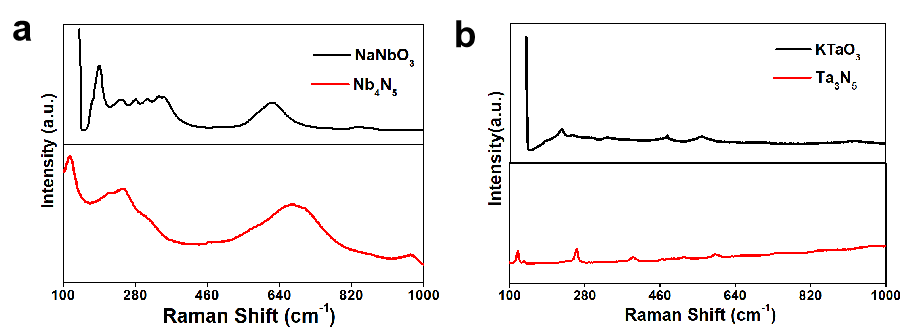


**Figure S5.** Raman image of PSC Nb_4_N_5_ and PSC Ta_3_N_5_. (a) Raman of the NaNbO_3_ and Nb_4_N_5_. (b) Raman of the KTaO_3_ and Ta_3_N_5_.


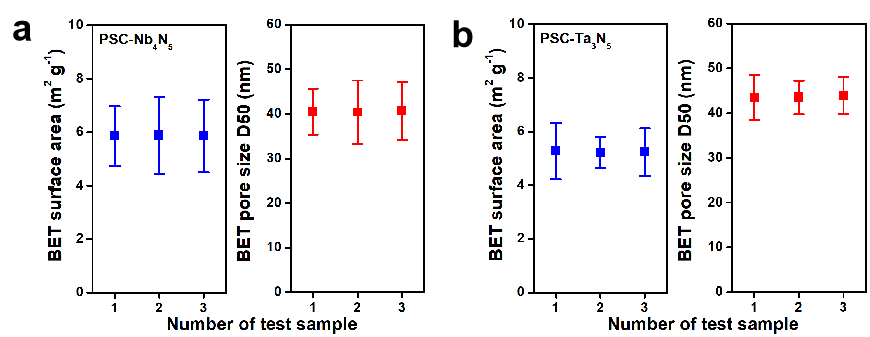


**Figure S6.** BET of PSC Nb_4_N_5_ and Ta_3_N_5_. (a, b) Surface area and pore size of PSC Nb_4_N_5_ and Ta_3_N_5_.


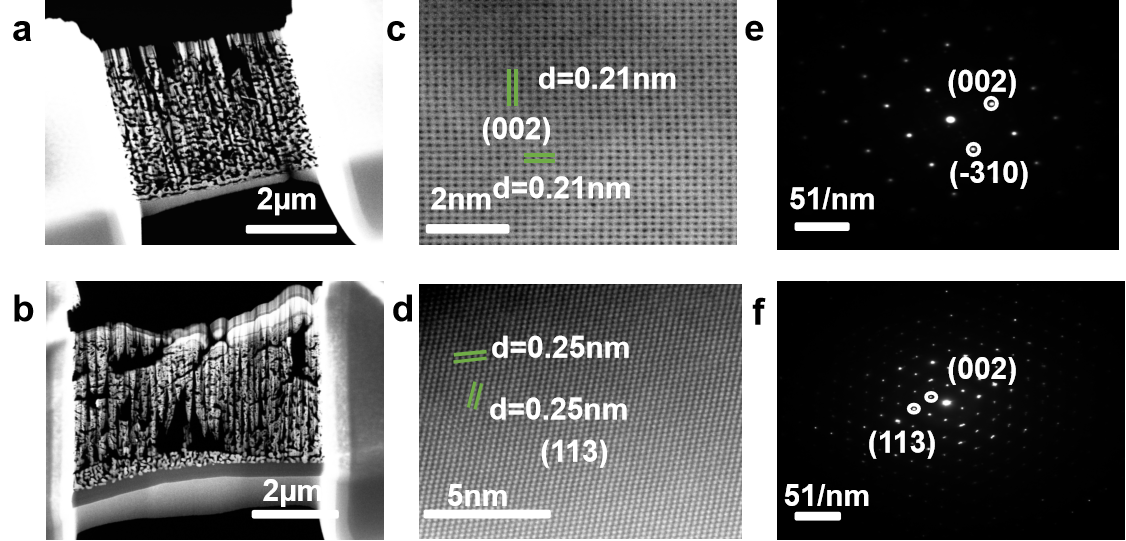


**Figure S7****.** STEM of PSC Nb_4_N_5_ and PSC Ta_3_N_5_. (a, b) Sample sections; (c, d) atomic arrangement distribution; (e, f) selected area electron diffraction (SAED).


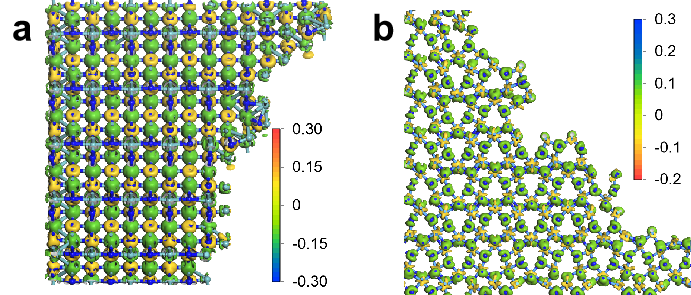


**Figure S8.** (a, b) Plot of electron density differences between PSC Nb_4_N_5_ and PSC Ta_3_N_5_ twisted surfaces.


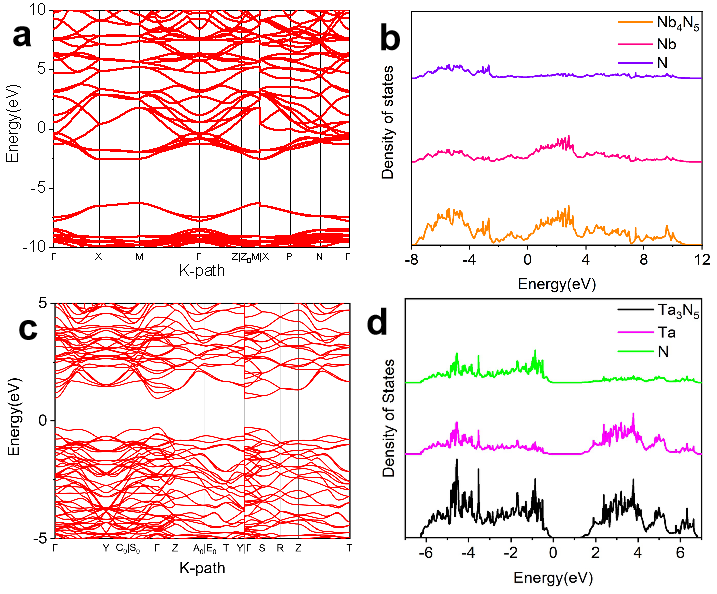


**Figure S9.** The band gaps and density of states of the (a, b) PSC Nb_4_N_5_ and (c, d) PSC Ta_3_N_5_.


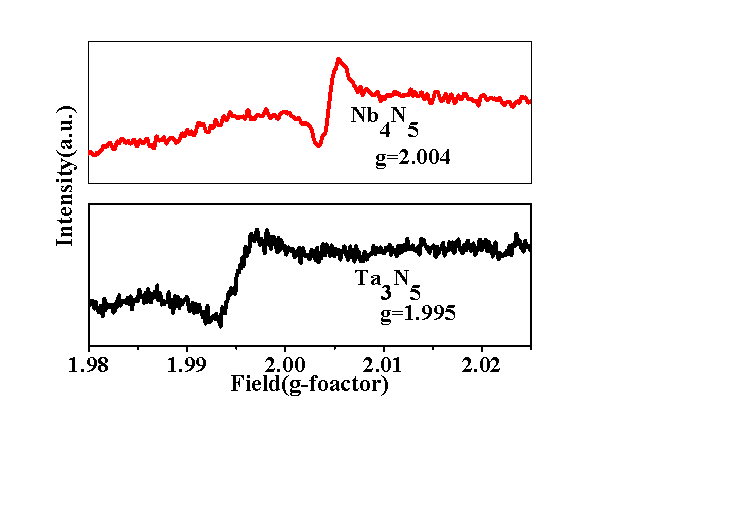


**Figure S10.** EPR diagrams of PSC Nb_4_N_5_ and PSC Ta_3_N_5_.


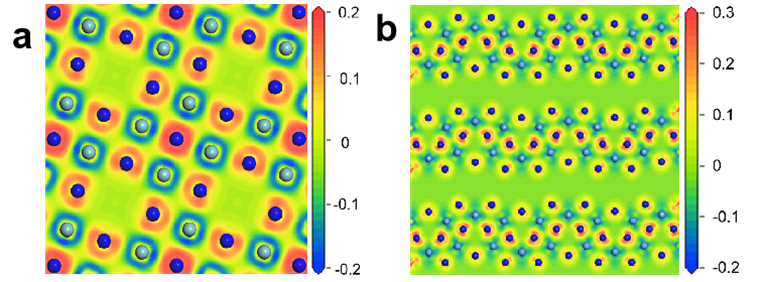


**Figure S11.** Differential charge density maps of the outermost surfaces of (a) PSC Nb_4_N_5_ and (b) PSC Ta_3_N_5_.

**
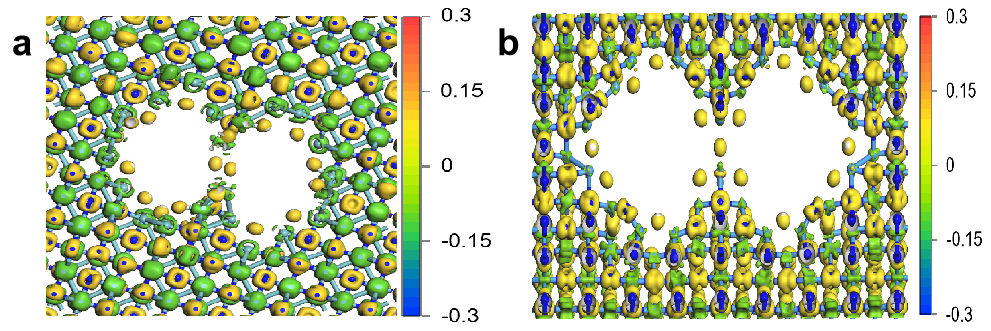
**

**Figure S12.** (a, b) Charge density plots of H adsorption on active Nb and Ta atoms in porous Nb_4_N_5_, Ta_3_N_5_ structures.

**
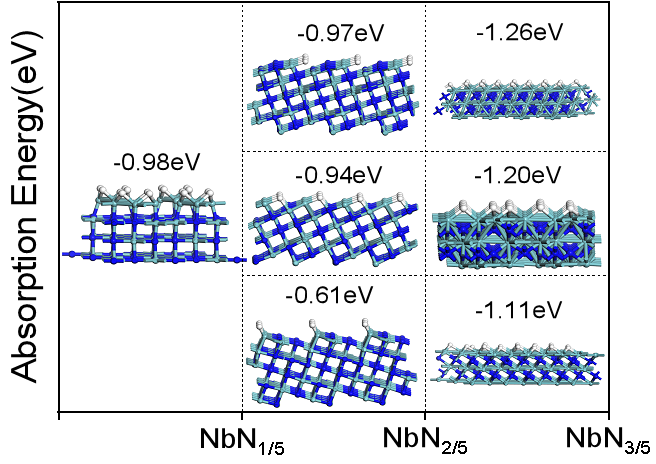
**

**Figure S13.** Adsorption energies for H adsorption on surfaces of different Nb-N configurations.


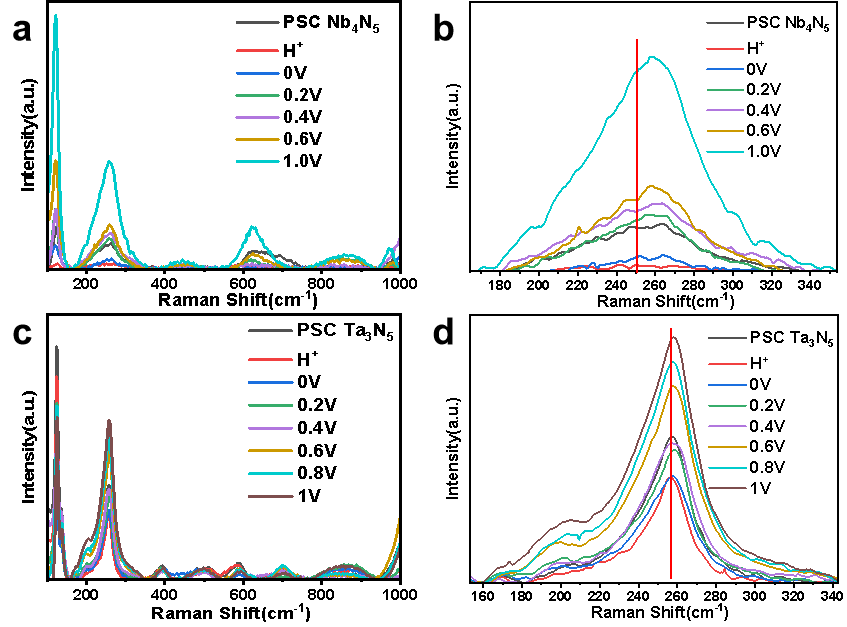


**Figure S14.** In situ Raman spectra at different voltages, (b, d) are localized magnified images of (a, b), respectively.


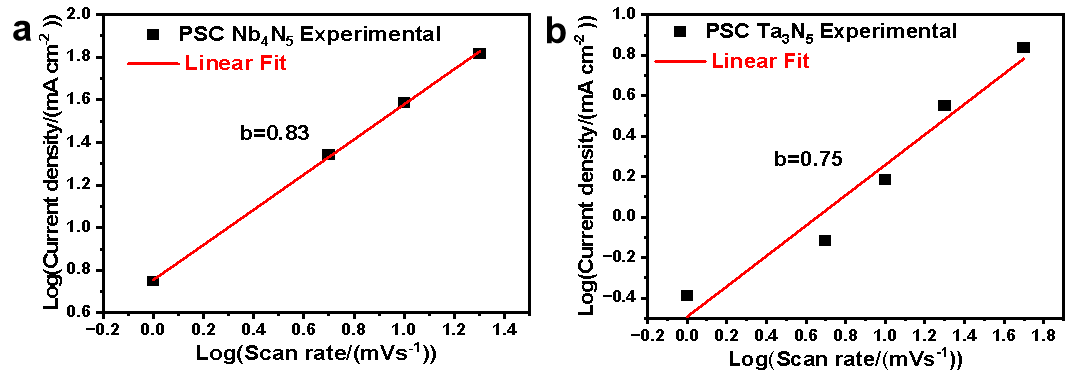


**Figure S15.** (a, b) The b-value of the CV curve current was determined with a CV scan range of 1-50 mV s^-1^.


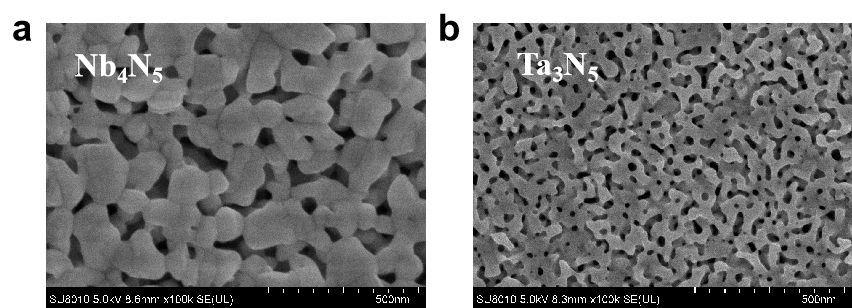


**Figure S16.** SEM images of PSC (a) Nb_4_N_5_ and (b) Ta_3_N_5_ after the long-term cycling test.

**Reference**

[1] G. Kresse, J. Furthmiiller, *Comp. Mater. Sci.* **1996**, 6, 15.

[2] J. P. Perdew, K. Burke, M. Ernzerhof, *Phys. Rev. Lett.* **1996**, 77, 3865.
